# Thrombotic Microangiopathy Post-COVID-19 Vaccination

**DOI:** 10.7759/cureus.60506

**Published:** 2024-05-17

**Authors:** Rahaf Salem, Ibrahim Al Mulla, Noure Alhouda, Junaid Iqbal, Giamal Gmati

**Affiliations:** 1 Department of Internal Medicine, King Abdulaziz Medical City, Riyadh, SAU; 2 Department of Medicine, King Fahad Medical City, Riyadh, SAU; 3 Department of Pathology and Laboratory Medicine, King Abdulaziz Medical City, Riyadh, SAU; 4 Division of Nephrology, Department of Medicine, King Abdulaziz Medical City, Riyadh, SAU; 5 Department of Oncology, Mid and South Essex NHS Foundation Trust, Chelmsford, GBR

**Keywords:** thrombosis, atypical hemolytic uremic syndrome, vaccine, thrombotic microangiopathy, covid-19

## Abstract

The emergence of COVID-19 has caused a wide spectrum of symptoms, ranging from asymptomatic to devastating symptoms, leading to death. One of the most serious complications of COVID-19 is the thromboembolic phenomenon, which has led to increased morbidity and mortality. Several vaccines were developed to protect against this infection and used widely across the globe. However, thromboembolic events were observed in the vaccinated population and were certainly the most commonly reported events following the COVID-19 vaccination. Although the thrombotic complications of COVID-19 were poorly understood, hyper-inflammatory responses were thought to be one of the main explanations for this infection sequel. In the setting of COVID-19 vaccines, there is still no clear understanding of the thrombosis pathophysiology, and, again, exaggerated pro-inflammatory and immune-mediated processes seem to be leading causes. Definitely, with the rise in reported cases of serious complications and increased awareness of these phenomena, we learn new theories and explanations that help us understand and manage those patients. We report the case report of two patients we managed over the last three years who presented with thrombotic microangiopathy following the COVID-19 vaccination.

## Introduction

The emergence of COVID-19/severe acute respiratory coronavirus 2 (SARS COV-2) in early December 2019 warranted the declaration of a pandemic in March 2020 by the World Health Organization. Despite the wide spectrum of symptoms caused by the virus, ranging from asymptomatic to devastating symptoms, leading to death, COVID-19 has led to an increase in morbidity and mortality rates and has been declared as a very high-risk global pandemic per the Coronavirus Disease (COVID-19) Situation Reports [1]. The COVID-19 infection has been associated with a high incidence of thrombosis and thromboembolic complications, and hyper-inflammatory response mediated by the immune system is considered a major factor in developing these complications [2]. As the high infectivity rates and fatalities were becoming more apparent, the need to develop a vaccine and specific viral treatment became a flashpoint for researchers.

The development of vaccines started as soon as the virus genome was identified in early January 2020. The sequencing of the viral genome led to mass vaccination. Four types of vaccines were developed against the COVID-19 virus: mRNA vaccines, non-replicative vector vaccines, inactivated vaccines, and subunit vaccines [3].

During the imminent process of vaccine development, their safety profile and effectiveness as well as adverse events were monitored and reported. Multiple vaccines were granted approval for emergency use. While the vaccines have been approved for Emergency Use Authorization (EUA), there is ongoing safety profile monitoring of all available vaccines.

Majority of side effects reported thus far are mild; however, rare and serious side effects such as cerebral venous sinus thrombosis (CVST), Guillain-Barré syndrome, thrombosis with thrombocytopenia syndrome (TTS), anaphylaxis, myocarditis, and pericarditis have also been reported [4].

A concern was raised in March 2021, when several thrombotic events related to the ChAdOx1-SARS-COV-2 (which is a non-replicative vector vaccine) were reported. Those events were in the form of disseminated intravascular coagulation (DIC), CVST, and hemorrhagic stroke. Despite the fact that 5.5 million people had received the vaccine at that time, these events were exceedingly rare to occur [3].

Even more rare complications related to vaccines have been reported, such as thrombotic microangiopathy (TMA). TMA is a syndrome characterized by clinical and pathological features. It can be further classified into hereditary and acquired. Clinical features include microangiopathic hemolytic anemia, thrombocytopenia, and organ injury, while pathological features suggest vascular damage as evident by arteriolar and capillary thrombosis [5].

Here, we describe two patients, both young men, who were treated at our institution in 2021. Both patients presented with a full-blown picture of TMA following COVID-19 vaccination.

## Case presentation

Case 1

A 29-year-old male, diagnosed with essential hypertension since the age of 24 years, presented to the Emergency Department (ED) with a four-day history of watery, non-bloody diarrhea. He had up to three to four episodes of diarrhea daily, which he associated with consuming grilled food four days prior to symptoms onset. He received his second dose of COVID-19 vaccine (mRNA-1273 vaccine) three days prior to his presentation. He developed fever and unilateral epistaxis post-SARS-CoV-2 vaccine, which resolved within 24 hours. He denied any history of fever, cough, headaches, joint pains, or myalgia. Similarly, he did not report any bleeding episodes, thrombotic events, skin rashes, or change in urine characteristics, and had no history of recent travel. He had no family history of thrombophilia or hematological disorders.

Clinical assessment was essentially unremarkable. His hemodynamics were stable, the patient was normotensive upon presentation to ED and euvolemic, and he had adequate urine output. The laboratory evaluation is shown in Table 1.

**Table 1:** Laboratory evaluation BUN, blood urea nitrogen; eGFR, estimated glomerular filtration rate; LDH, lactate dehydrogenase; C3, complement 3; C4, complement 4

Lab Test	Result	Reference Range
Hemoglobin	79gm/L	135-180 gm/L
White blood cells	6.1 10^9^/L	4-11 x 10^9^/L
Platelets	100 x10^9^/L	150-400 x 10^9^/L
Creatinine	1416 umol/L	64-110 umol/L
BUN	37.5 mmol/L	3.2-7.4mmol/L
eGFR	4 mL/min/1.73m^2^	60 mL/min/1.73m^2^
LDH	566 U/L	125-220 U/L
Homocysteine	23.0 umol/L	5.5 -16.2 umol/L
C3	0.785 g/L	0.79-1.52 g/L
C4	0.366 g/L	0.16-0.38 g/L

**Figure 1:**
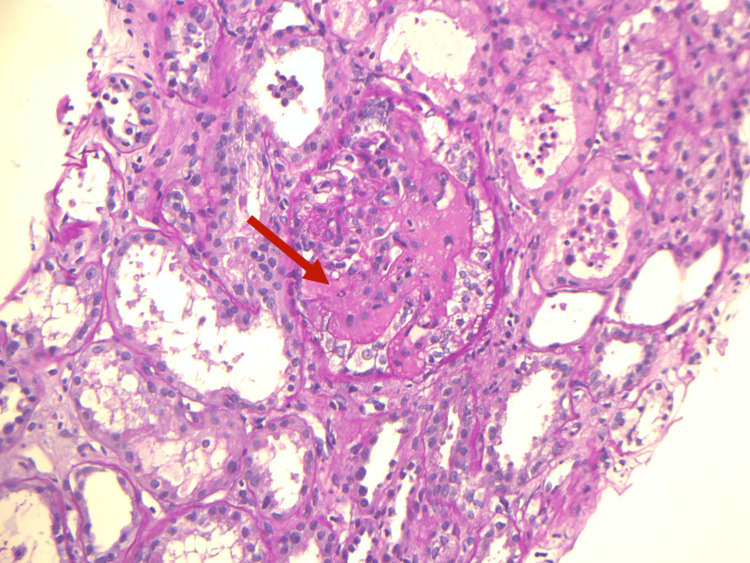
Kidney biopsy showing fibrin thrombi in the glomerulus (arrow) and acute tubular injury

**Figure 2:**
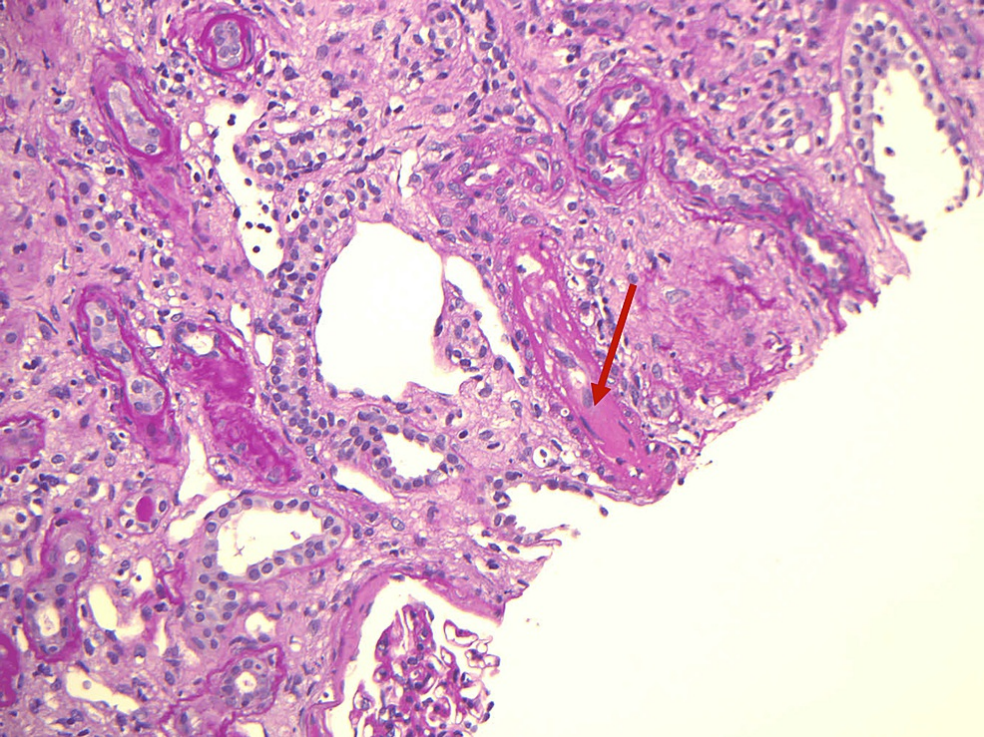
Kidney biopsy showing arteriolar fibrin thombi (arrow)

Laboratory evaluations for atypical hemolytic uremic syndrome (aHUS) including complement regulatory protein mutation were all negative. Urine homocysteine was within normal limits, and stool culture did not isolate any pathogens. Peripheral blood smear showed 3.7% schistocytes. Hemolysis panel confirmed ongoing non-immune hemolysis. These findings were suggestive of microangiopathic hemolytic anemia (MAHA). ADAMTS13 (a distntegrin and metalloproteinase with a thrombospondin type 1 motif, member 13) activity, also known as von Willebrand factor-cleaving protease, antigens, and antibodies were all within normal range. Renal ultrasound was unremarkable with normal-sized kidneys and no evidence of renal vascular disease. Abdominal and pelvic CT scan revealed bilateral non-obstructive renal stones with no peri-nephric collection. Kidney biopsy demonstrated a classical picture of chronic TMA in the form of glomerular basement membrane (GBM) thickening, replication and focal mesengiolysis, with focal fibrin thrombi formation, and arteriolar endothelial swelling with onion skinning and mucoid changes, as demonstrated on light microscopy (Figures 1, 2). In addition, there was a presence of advanced chronic kidney disease with severe global and segmental glomerulosclerosis.

Owing to his clinical and biochemical presentation and the presence of severe stage 3 acute kidney injury (AKI) with reduced urine output, he required urgent hemodialysis. The hematology team was consulted, and plasma exchange (PE) treatment was recommended. Daily monitoring of complete blood count (CBC), hemolysis panel, and schistocytes count in peripheral blood smear was performed. All parameters of hemolytic anemia significantly improved, and platelet count recovered following the first session of PE. The patient underwent daily PE sessions with fresh frozen plasma in a 1:1 ratio for the first three days and then every other day for a total of 14 sessions, after which he was successfully weaned off PE sessions. However, the patient’s kidney function failed to recover, and he remained hemodialysis-dependent at the time of discharge from the hospital.

Case 2

A 40-year-old male, generally fit and well, presented to the ED with progressive dyspnea and bilateral peri-orbital puffiness, occurring seven days following first dose of ChAdOx1-SARS-COV-2 vaccine.

He had no fever, cough, headaches, joint pains, or myalgia. There was no change in urine output, frequency, color, or frothiness. There were no associated symptoms of abdominal pains, gum bleeding, bleeding per rectum, or any rash. He neither had a prior history of hematological disorder or kidney disease nor he had travelled out of city recently. Family history was unremarkable. Apart from moderate periorbital and bilateral ankle edema, clinical assessment at admission was essentially unremarkable with normal and stable vital signs (heart rate of 88/min, blood pressure of 169/70 mmHg, and oxygen saturation 99% on room air) and he appeared mildly dehydrated. Initial laboratory evaluation is shown in Table 2.

**Table 2:** Laboratory evaluation BUN, blood urea nitrogen; eGFR, estimated glomerular filtration rate; LDH, lactate dehydrogenase; C4, complement 4

Lab Test	Result	Reference Range
Hemoglobin	83 g /L	135-180 g/L
White Blood Cell	5.71 x 10^9^/L	4-11 x 10^9^/L
Platelets	152 x 10^9^/L	150-400 x 10^9^/L
Creatinine	960 umol/L	64-110 umol/L
BUN	43.8 mmol/L	3.2-7.4 mmol/L
eGFR	6 mL/min/1.73m^2^	60 mL/min/1.73m^2^
LDH	628 U/L	125-220 U/L
C4	0.384 g/L	0.16-0.38g/L

Laboratory evaluations for aHUS including complement regulatory protein mutation were all negative. Urine homocysteine was within normal limits. Stool culture did not isolate any organism. Renal ultrasound showed normal kidneys.

On the second day of admission, the patient’s hemoglobin and platelet count dropped to 6.4 mg/dL and 130 x 10^3^/mcL, respectively Peripheral blood smear showed 2-3% schistocytes. Hemolysis panel was positive and consistent with non-immune hemolysis. These findings were suggestive of MAHA. ADAMTS13 activity, antigens, and antibodies were all within normal range.

**Figure 3:**
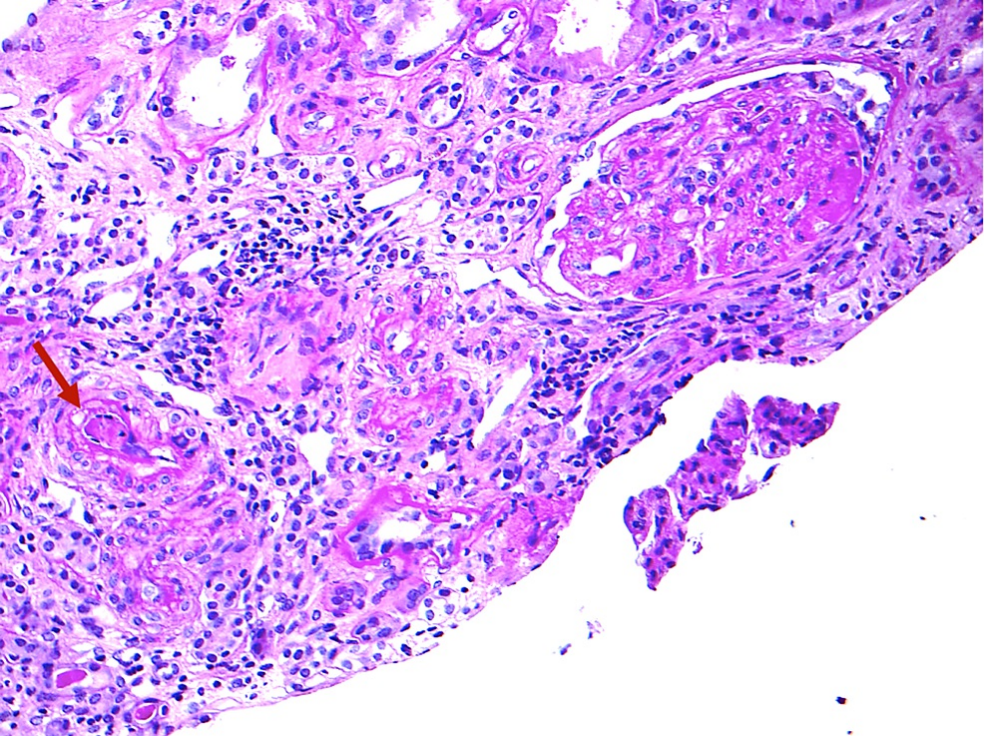
Mark chronic changes with arteriolar thrombus noted (red arrow), and the glomerulus show GBM thickening, replication, and segmental scarring consistent with chronic TMA changes (PAS, 10x) TMA, thrombotic microangiopathy; PSA, periodic acid-Schiff

A diagnostic native kidney biopsy showed microthrombi in the arterioles, and glomerular capillaries with fibrinoid necrosis of blood vessel walls were also noted. There was evidence of chronic kidney damage with moderate interstitial fibrosis and tubular atrophy (IFTA) and a large number of sclerosed glomeruli (Figure 3).

Considering severe stage 3 oliguric AKI, the patient was commenced on hemodialysis. He received intravenous methylprednisolone 1 mg/kg daily for three days. He received daily PE; however, PE had to be discontinued after two sessions due to perinephric hematoma as a complication on renal biopsy. Rituximab 375 mg/m^2^ as an alternative to PE was commenced on a weekly basis for total of four doses. Daily monitoring of CBC, hemolysis panel, and schistocytes on peripheral blood smear showed recovery within few days of PE and rituximab. However, the renal function failed to improve, and the patient remained dialysis-dependent.

## Discussion

It has been established that the ChAdOx1 nCoV-19 adenoviral vector vaccine against SARS-CoV-2 is associated with vaccine-induced immune thrombocytopenia and thrombosis (VITT) and other possible hematological phenomena, such as development of de novo complement mediated hemolytic or an exacerbation of pre-existing complement mediated hemolytic anemia [6].

TMA are a group of disorders characterized by the presence of MAHA, thrombocytopenia, and end-organ capillary thromboses. TMAs are a syndrome of capillary and arteriolar thrombosis that can have a variety of causes. There are few suggestive mechanisms on how the COVID-19 virus can cause TMAs, such as direct viral toxicity to vascular endothelium, microvascular thrombosis, and complement-mediated disorder. However, thrombotic thrombocytopenic purpura (TTP), Shiga toxin mediated TMA, or complement-mediated TMA are most common apart from DIC. The definitive diagnosis of any of the above requires testing that may not always be readily available. This includes ADAMTS13 activity and inhibitor assay for TTP, next-generation DNA sequencing, and aHUS complement panel [7]. TMAs can also occur in a variety of other clinical conditions such as following hematopoietic and solid organ transplantation, pregnancy, underlying malignancy, glomerular diseases, autoimmune disorders, and with the use of certain drugs [8].

Since the identification of the COVID 19 virus, efforts to develop an effective vaccine against SARS-CoV-2 began to reduce morbidity and mortality and to mitigate consequent global health and economic impact. Similar to the development of any new medication or vaccine, its safety, efficacy, and adverse effects were closely monitored. As the number of available vaccines increased throughout the world, serious adverse effects were observed following immunization. These include TMA, TTS, and VITT [9].

Few cases of COVID-19 infection associated with renal TMA have been reported. In a case series from French and Swiss renal transplantation and internal medicine units, none of whom had personal or familial history of aHUS, three out of the five patients were renal transplant recipients and were on immunosuppressive therapy, all five patients had mild respiratory symptoms of COVID-19, and only one patient required low-grade oxygen therapy. Kidney biopsy was performed in all patients; and there were no significant immune deposits. All patients had detectable ADAMTS13 activity. The time interval between the diagnosis of COVID-19 infection and TMA diagnosis ranged from 0 to 30 days. They concluded that COVID-19 is a potential newly identified trigger for complement-mediated aHUS [10].

The proposed pathophysiology is the activation of the proximal complement through the alternative, classical, and lectin pathways. These three pathways lead to C3 activation and C3 convertase formation. The activated C3 amplifies this response, leading to C3 fragment deposition on target cells. In the presence of increased surface density of deposited C3b, the terminal (lytic) pathway is triggered, causing the formation of C5b-9 or membrane attack complex (MAC) on the surface of target cells. Endothelial dysfunction and microvascular thrombosis result from persistent complement activation. Similarly, it is proposed that coronaviruses proteins bind to a key protein of the lectin pathway (MASP-2 [Mannan-binding lectin serine protease 2]), leading to complement-mediated inflammatory lung injury [11].

Our first patient presented within a week of COVID-19 vaccination, in concordance with average timeline reported in the literature, i.e., 5-30 days (median of 4-16 days), post-SARS-CoV-2 immunization [3,12]. The initial abnormalities of acute kidney injury and MAHA evident by the presence of schistocytes and thrombocytopenia were suggestive of a picture of aHUS.

There is a series of similar cases presenting in a similar fashion shortly after COVID 19 vaccine, supporting the observation that TMA is associated with SARS-Cov-2 immunization. This observation, however, requires further study to confirm SARS-CoV-2 vaccine as a trigger for HUS/TMA. Similar to our patient, most TTS cases were individuals aged 20 to 50 years; however, it is more common in females [12].

In concurrence with the recommendations from the American Society of Hematology and the Expert Hematology Panel (EHP) for the British Society for Haematology, our patients, like other cases reported, were managed with high-dose glucocorticoids, PE, and hemodialysis sessions. Other patients also received intravenous immunoglobulin (IVIG) [13].

In our second patient, SARS-CoV-2 vaccination was the most likely trigger of TMA, as he presented with progressive dyspnea and bilateral periorbital puffiness, which occurred seven days after receiving the first dose of ChAdOx1 nCoV-19 adenoviral vector vaccine, and clinical and laboratory findings were suggestive of TMA, which was later confirmed with kidney biopsy. He was managed with steroids and PE and eventually required to continue to long-term hemodialysis as his kidney function did not recover.

A case has been reported in which a patient presented with TMA and rhabdomyolysis four weeks after receiving the first dose of mRNA-1273 vaccine. He was commenced on argatroban initially, followed by anticoagulation with heparin, hemodialysis, high-dose methylprednisolone, and IVIG. However, he developed massive bleeding in the gastrointestinal tract and iliopsoas muscle; thus, anticoagulation was held, and eventually the patient was started on eculizumab. The patient died after 18 days of admission. An autopsy was performed, which confirmed findings consistent with TMA in the kidney [14].

There are several concerns that need to be addressed in regard to managing and possibly preventing TMAs post-ChAdOx1 nCoV-19 vaccine. The exact mechanism and the in-risk population are hard to identify, and further data are required to find a correlation between ChAdOx1 nCoV-19 vaccine and TMAs.

As vaccines against COVID-19 are becoming more popular and the need for booster doses is being carefully reviewed, it is essential to recognize and report the possible serious although rare side effects.

In conclusion, our report sheds light on the need to further report and discern for similar presentations and to screen for primary causes of TMA to be able to correctly measure the risk of vaccine-associated TMA events.

Similar to all aspects in medicine, drawing attention to the rare yet possible complications that might result from an intervention is vital as it will enable physicians and clinical practitioners to vigilantly monitor for any of the side effects. Immediate action is warranted when such complications are identified, which might be life-saving.

As in our reported two patients, reaching an accurate diagnosis in a timely manner has a positive impact on their outcome and provides further healthcare and recommendations to avoid further deterioration of their clinical condition and any further complications.

Although our patients have not fully made a recovery of their renal function in the short-term follow-up we have had, other patients might regain function fully and lead normal lives if acted upon early in their treatment course.

## Conclusions

Like all aspects of medicine, drawing attention to the rare yet possible complications that might result from an intervention is vital. This will enable physicians and clinical PR actioners to vigilantly monitor for any side effects. Immediate action is warranted when such complications are identified, which could be lifesaving.

TMA post-COVID-19 vaccination is a well-recognized complication and carries a drastic sequela if not identified early. As discussed earlier, reaching an accurate diagnosis in a timely manner has a positive impact on the outcome. It also allows for the provision of further health care and recommendations to avoid further deterioration of their clinical condition and any complications. In conclusion, our report sheds light on the need for further investigation, understanding of similar presentations, and screening for primary causes of TMA. This will enable us to accurately measure the risk of vaccine-associated TMS events.
